# Case Study: Mechanism for Increased Follicular Helper T Cell Development in Activated PI3K Delta Syndrome

**DOI:** 10.3389/fimmu.2019.00753

**Published:** 2019-04-12

**Authors:** Timothy J. Thauland, Laurence Pellerin, Robert S. Ohgami, Rosa Bacchetta, Manish J. Butte

**Affiliations:** ^1^Division of Immunology, Allergy, and Rheumatology, Department of Pediatrics, University of California, Los Angeles, Los Angeles, CA, United States; ^2^Division of Pediatric Stem Cell Transplantation and Regenerative Medicine, Department of Pediatrics, Stanford University, Stanford, CA, United States; ^3^Department of Pathology, Stanford University, Stanford, CA, United States

**Keywords:** primary immunodeficiency, activated PI3K delta syndrome (APDS), PI3K, T follicular helper cells, osteopontin

## Abstract

Gain-of-function variants in p110δ, the catalytic subunit of phosphatidylinositol 3-kinase (PI3K) expressed in lymphocytes, cause activated PI3-kinase δ syndrome (APDS), a primary immunodeficiency that is characterized by recurrent infections, viremia, lymphadenopathy, and autoimmunity. The mechanism of autoimmunity in APDS has not been well-understood. Here, we show the profound skewing of peripheral CD4^+^ T cells to a T follicular helper (T_FH_) phenotype in a patient with APDS bearing a novel p110δ variant, Y524S. We also saw a diminishment of transient Foxp3 expression in activated T cells. Mechanistic studies revealed that both the new variant and a previously described, pathogenic variant (E81K) enhanced an interaction between intracellular Osteopontin and p85α. This interaction had been shown in mice to promote T_FH_ differentiation. Our results demonstrate a new influence of PI3K on human T cell differentiation that is unrelated to its lipid-kinase activity and suggest that T_FH_ should be monitored in APDS patients.

## Introduction

Activated PI3-kinase δ syndrome (APDS), also known as p110δ-activating mutation causing senescent T cells, lymphadenopathy and immunodeficiency (PASLI), is a primary immunodeficiency disease (PID) caused by gain-of-function variants in *PIK3CD*. This gene encodes the δ isoform of the p110 catalytic subunit of phosphatidylinositol 3-kinase (PI3K). APDS is characterized by recurrent respiratory infections, chronic herpes virus infection, autoimmunity, splenomegaly, and lymphadenopathy (1–5). Many patients also have diminished total and naïve CD4^+^ cells (2) and an increase in senescent CD8^+^ effector cells (6–8). Multiple cases of primary sclerosing cholangitis have been observed in one family with a common APDS variant (9), and B cell lymphomas have also been noted (4, 8, 10).

T cell blasts from APDS patients show increased Akt phosphorylation both at baseline and after stimulation (2, 3). Positron-emission tomography scans have shown increased glucose uptake in the secondary lymphoid organs of patients with APDS (2, 11) and improper activation of PI3K-Akt-mTOR pathway explains the major clinical findings in APDS: T cell proliferation causes lymphadenopathy, decreased naïve T cells and increased memory and effector cells. This inappropriate proliferation ultimately leads to senescence, resulting in frequent infections. Patients treated with rapamycin, which inhibits the mTOR pathway, have shown improvement (2).

Lucas et al. identified APDS-causing variants in three different domains of p110δ. The high sequence similarity between p110δ and p110α allowed the APDS-causing variants to be mapped to the corresponding oncogenic variants in p110α, which have been studied extensively (2). On the basis of these studies, pathogenic variants in the membrane-binding C2 domain (N334K) and the helical domain (E525K) were hypothesized to disrupt inhibitory contacts between p110δ and p85α, while a pathogenic variant in the kinase domain (E1021K) most likely enhances association with the plasma membrane.

Interestingly, an APDS-like disease caused by splice-site variant in the p85α gene resulting in the deletion of an exon from the inter-SH2 domain has also been described (12, 13). This deletion reduced the association between p110δ and p85α in immunoprecipitation experiments (12). The dissociation of p85α from p110δ may have functional consequences in these patients, aside from the enzymatic gain-of-function of p110δ. Recently, experiments in mice have shown that ICOS signaling results in the binding of p85α to non-secreted, intracellular osteopontin (iOPN) (14). The p85α-iOPN interaction was translocated to the nucleus where iOPN stabilized Bcl-6, the master transcription factor for T follicular helper (T_FH_) cell differentiation (14). Thus, destabilizing APDS variants could enhance T_FH_ differentiation.

APDS patients with an E1021K and E525A in PIK3CD have been shown to uniformly possess large numbers of T_FH_ cells in their peripheral blood (11). A recent study has confirmed these findings and proposed a mechanism linking the inappropriate phosphorylation of FOXO1 to the T_FH_ phenotype in APDS patients (15). The over-abundance of T_FH_ cells in APDS patients likely leads to defects in observed defects in humoral immunity (15, 16). PI3K activity leads to inactivation of FOXO1 via phosphorylation by Akt, and mice lacking FOXO1 have enhanced T_FH_ differentiation (17). Further, T cells from mice heterozygous for a PIK3CD allele with the E1021K variant knocked in showed enhanced T_FH_ differentiation upon immunization (15).

The phosphatase PTEN opposes the activity of PI3K by dephosphorylating PIP3 at the plasma membrane (18). Perhaps not surprisingly, patients with loss-of-function, pathogenic variants in PTEN have an APDS-like syndrome (11). Like APDS patients, PTEN-deficient patients display enhanced phosphorylation of Akt, mTOR, and S6 in their T cells, highlighting the importance of these opposing enzymes in maintaining the appropriate level of Akt activity. Interestingly, although patients with pathogenic variants in PTEN phenocopy the heightened activity in the Akt pathway, they do not have enhanced numbers of T_FH_ cells (11). These data strongly suggest that enhanced activation of Akt (and phosphorylation of FOXO1) may not solely drive the T_FH_ phenotype observed in APDS patients. Thus, we sought another mechanism beyond the PI3K-PTEN axis that drives T_FH_ differentiation.

We identified a young girl whose presentation was consistent with APDS: recurrent sinopulmonary infections, CMV viremia, and severe lymphadenopathy. Sequencing revealed a novel variant—Y524S—in the helical domain of p110δ at the interface between p110δ and p85α. Significantly, the patient had large numbers of T_FH_ cells in peripheral blood, and lymph nodes revealed follicular hyperplasia. Mechanistically, expression of pathogenic p110δ containing APDS variants resulted in increased association between p85α and iOPN *in vitro*. These results demonstrate that, in addition to lymphoproliferation-associated symptoms, APDS patients should be monitored for defects in CD4^+^ T cell differentiation.

## Results

A 7-year old girl with a history of serious infections was referred to our center for worsening lymphadenopathy. At 3 years of age, she was diagnosed with disseminated CMV viremia and was successfully treated with valganciclovir. At 4 years of age, an immunology workup revealed normal responses to tetanus, diphtheria and influenza vaccines but a failure to respond to pneumococcal vaccination, as well as, high IgM and low IgG levels. Her lymphadenopathy was first noted at age 11 months and had become generalized by age 3. Multiple biopsies demonstrated reactive lymph nodes with both follicular and paracortical hyperplasia, but no evidence of malignancy was detected. Treatment with steroids was unsuccessful, however, two courses of rituximab, at ages 6 and 7, completely, but temporarily, resolved her lymphadenopathy.

**Figure 1:**
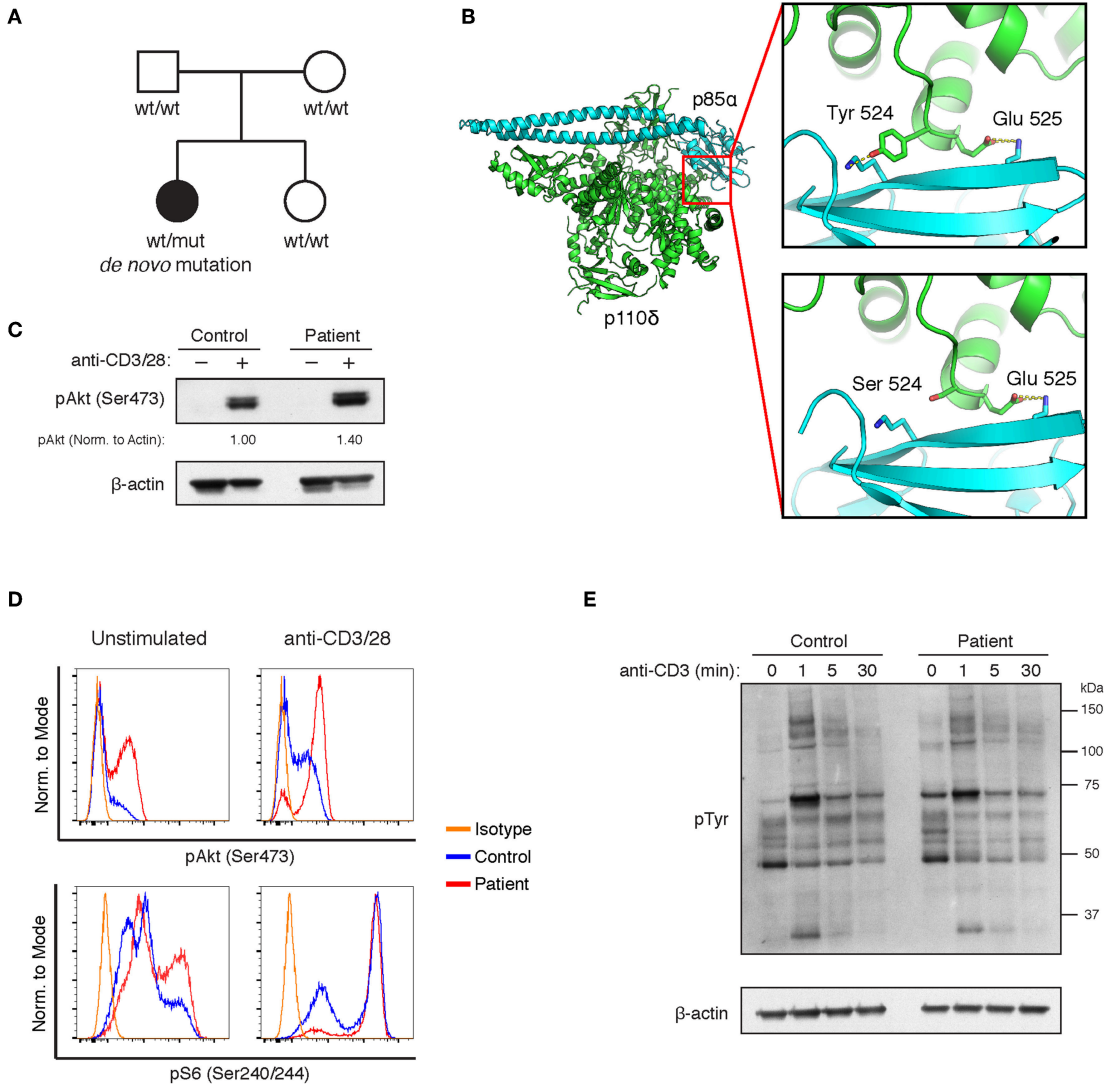
A novel Y524S variant in p110δ causes APDS. **(A)** Pedigree showing a *de novo* variant in p110δ. **(B)** Molecular model showing the location of the Y524S variant in relation to p85α. Note the loss of the hydrogen bond and buried surface area when Tyr 524 is mutated to Ser. **(C)** Levels of phospho-Akt (Ser473) and β-Actin in CD4 cells purified from control or patient PBMCs were assayed by Western blotting. Cells were unstimulated (−) or stimulated with anti-CD3 and anti-CD28 for 5 min (+). Results are representative of three experiments. **(D)** Flow cytometry of control or patient CD4^+^ PHA blasts. Cells were assayed for phospho-Akt (Ser473) and phospho-S6 (Ser240/244) with or without stimulation for 10 min with anti-CD3 and anti-CD28. Results are representative of two experiments. **(E)** Western blotting for phosphotyrosine in freshly purified control or patient CD4^+^ T cells, either unstimulated or stimulated for the indicated times with anti-CD3.

As shown in Table 1, the patient's T cell compartment was characterized by low proportions of naive CD4^+^ cells and high proportions of CD4^+^ and CD8^+^ memory cells. The absolute number of B cells was low, with a low percentage of memory B cells and a very high percentage of transitional B cells. Given her clinical course, we suspected that the patient suffered from a primary immunodeficiency. Whole exome sequencing revealed a novel, *de novo* variant in exon 13 of p110δ, NM_005026.4:c.1571A>C (g.9780849 (chr1, hg19)) (Figure 1A). The variant was verified by Sanger sequencing. This missense variant results in a p.Y524S substitution in the helical domain of p110δ. The helical domain interacts with the nSH2 domain of the inhibitory subunit p85α, and Y524 lies on the surface of p110δ directly adjacent to another APDS-causing variant (E525K) (Figure 1B). The variant alters the inter-molecular hydrogen bonding network with p85α and reduces buried surface area. Thus, we reasoned that our patient's variant most likely weakens association of p110δ with p85α, resulting in inappropriate PI3K activity.

**Table 1:** Lymphocyte phenotyping.

	**Patient I**		**Patient II.A**		**Patient II.B**	
Age (years)	9		14		19	
**CD4**^**+**^ **T cells**		**Ref. Range**		**Ref. Range**		**Ref. Range**
CD4^+^ cells/microliter	**590**	650–1,500	**340**	530–1,300	**284**	530–1,300
CD4^+^CD45RA^+^ naïve T cells (%)	**33**	46–77	39	33–66	**27**	33–66
CD4^+^CD45RO^+^ memory T cells (%)	**60**	13–30	**59**	18–38	**72**	18–38
CD4^+^HLA-DR^+^ activated T cells (%)	**21**	3–13				
**CD8**^**+**^ **T Cells**						
CD8^+^ cells/microliter	**1,728**	370–1,000	**175**	330–920	518	330–920
CD8^+^CD45RA^+^ naïve T cells (%)	65	63–92	66	61–91	**54**	61–91
CD8^+^CD45RO^+^ memory T cells (%)	**24**	4–21	**29**	4–23	**35**	4–23
CD8^+^HLA-DR^+^ activated T cells (%)	17	6–29				
**B Cells**						
CD19^+^ cells/microliter	**69**	300–600	410	200–600	136	100–600
CD27^+^ memory B cells (%)	**6**	18.6–46.7	**3.3**	13.3–47.9	**1.5**	17.5–46.5
CD38^+^IgM^+^ transitional B cells (%)	**74**	4.6–8.3				
CD21^low^ immature B cells (%)	**12.3**	5.9–25.8	**58.5**	2.9–13.2	15.4	3.2–19.6
**Immunoglobulins**						
IgM (mg/dL)	**1,230**	53–334	**199**	40–140	**320**	44–277
Total IgG (mg/dL)	**486**	613–1,295	**1,550**	650–1,500	1,410	726–1,521
IgA (mg/dL)	**9**	69–309	143	60–310	214	87–426

*Values outside of the reference range are in bold. Reference range values are from Shearer et al. (19) for T cells and Piatosa et al. (20) for B cells*.

Activation of the PI3K pathway leads to Akt phosphorylation. Other APDS-causing variants, including E525K, have been shown to increase Akt phosphorylation both basally and after TCR stimulation (2, 3). Akt phosphorylation was enhanced in freshly purified CD4^+^ cells from the patient upon stimulation, however, basal phospho-Akt levels were not different than controls (Figure 1C). Basal pAkt is typically increased in T cell blasts from APDS patients (2). Thus, we established PHA blasts from the patient's PBMCs and compared phospho-Akt and phospho-S6 levels to controls. Enhanced phosphorylation of Akt and S6 was apparent, regardless of activation (Figure 1D). We also examined TCR signaling by stimulating CD4^+^ T cells with anti-CD3ε mAb and assaying phosphotyrosine levels by Western blot. The patient's T cells responded similarly to controls (Figure 1E). These results show that the Y524S variant increases PI3K activity in a similar fashion to other APDS variants.

**Figure 2:**
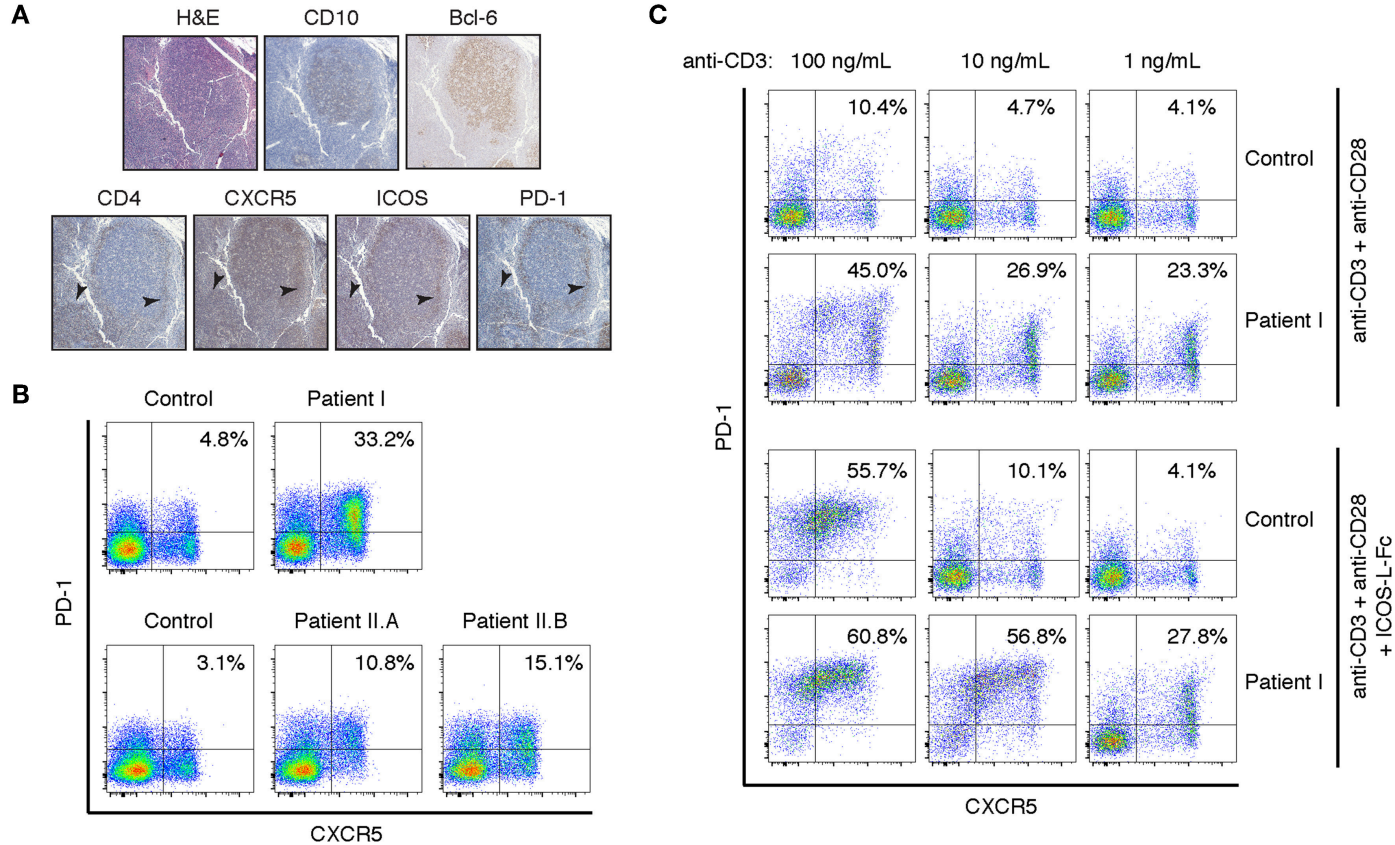
Peripheral and lymph node CD4^+^ T cells have a T_FH_ phenotype in the Y524S APDS patient. **(A)** Serial sections of a lymph node biopsy from the patient stained for H&E, CD10, CD4, Bcl-6, CXCR5, ICOS, and PD-1. Arrowheads indicate areas of CD4^+^ cells that are also positive for CXCR5, ICOS, and PD-1. **(B)** FACS analysis of CXCR5 and PD-1 on CD4^+^ T cells from fresh control and patient PBMCs. **(C)** CXCR5 and PD-1 expression on activated T cells. Freshly isolated CD4^+^ T cells from control and Patient I were stimulated for 4 days with the indicated concentrations of plate-bound anti-CD3, with or without 2 ug/mL ICOS-L-Fc. Anti-CD28 was included at 1 ug/mL in all cultures. Results are representative of two experiments.

Staining of lymph node biopsies for CXCR5, ICOS, and PD-1 revealed intense staining in CD4^+^ T cells surrounding CD10^+^Bcl-6^+^ germinal centers (Figure 2A). In comparison, a reactive lymph node from a subject without primary immunodeficiency has scattered PD-1+ T cells stained in the germinal center but not significantly in the band of lymphocytes that surround the germinal center (Figures S2A,B). In agreement with recent results from APDS patients bearing variants at E525 or E1021 (11), peripheral CD4^+^ T cells also had a circulating T_FH_ phenotype. More than 30% of peripheral CD4^+^ T cells were CXCR5^+^PD-1^+^, compared to approximately 5% in a healthy control (Figure 2B). Given that APDS-causing variants in both the helical (E525K and Y524S) and kinase (E1021K) domains enhance T_FH_ differentiation, we wondered whether N-terminal APDS variants also result in accumulation of T_FH_ cells in the periphery. To that end, we examined two siblings (Patient II.A and Patient II.B) with an E81K variant in the ABD domain of p110δ (Table 1). Patient II.A has been previously described [Patient B.1 in Ref. (21)], and prior to this study was the only APDS patient identified with an E81K variant. We found that both patients with the pathogenic E81K variant had increased peripheral T_FH_ cells, albeit to a lesser degree than Patient I (Figure 2B).

To assess the tendency to undergo differentiation to T_FH_, we stimulated CD4^+^ T cells with a range of anti-CD3 mAb concentrations and anti-CD28 mAb, in the presence or absence of ICOS ligand (ICOS-L). Both control and patient cells maintained their T_FH_ phenotype when offered sub-optimal levels of stimulation and roughly doubled the percentage of CXCR5^+^PD-1^+^ cells after 4 days of stimulation with a high concentration of anti-CD3 in the absence of differentiation factors (Figure 2C). This increase likely represents increased receptor expression among previously differentiated cells upon stimulation. In the presence of ICOS-L, control and patient cells differentiated to a similar extent upon high stimulation, while maximal differentiation occurred at 10-fold lower levels of stimulation in the patient's cells (Figure 2C). Thus, the higher basal signaling in APDS p110δ variants may act synergistically with receptor-mediated PI3K signaling to drive T_FH_ differentiation upon sub-optimal stimulation.

**Figure 3:**
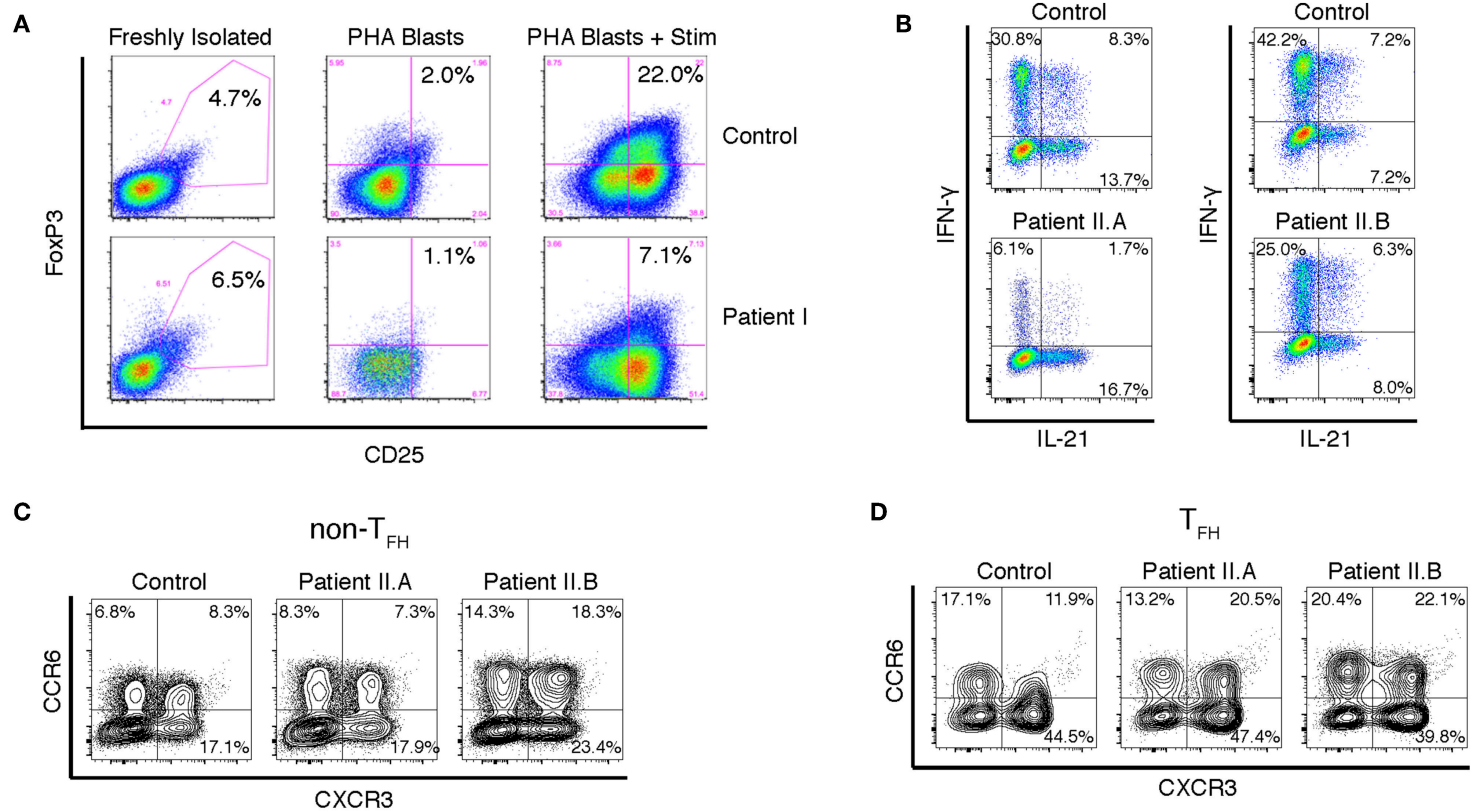
Altered FOXP3 expression and cytokine production in T cells from APDS patients. **(A)** FACS analysis of CD25+FOXP3+ Treg cells in freshly isolated PBMCs and in PHA blasts with or without restimulation with anti-CD3/28 beads and IL-2. Results are representative of three experiments. **(B)** Intracellular staining of CD4^+^ T cell blasts stimulated with PMA and ionomycin. **(C,D)** FACS analysis of chemokine receptors on CD4^+^ cells from freshly isolated PBMCs. CXCR5- non-Tfh cells **(C)** and CXCR5+PD-1+ Tfh cells **(D)** were assayed for CXCR3 and CCR6 expression.

Because signaling through PI3K negatively regulates FoxP3 expression and Treg induction (22, 23), we examined whether Patient I had a normal frequency of peripheral CD4^+^CD25^+^FoxP3^+^ Treg cells and whether FoxP3 was normally upregulated in CD4^+^ T cells upon TCR mediated activation. The percentage of peripheral CD4^+^CD25^+^FoxP3^+^ Tregs was normal in the patient, but PHA blasts from Patient I were impaired in their ability to express FoxP3 upon stimulation (Figure 3A). These results suggest that lymphoproliferation and autoimmunity in these patients is due to impaired cell intrinsic regulation of proliferation rather than to lack of Treg cells. However, we cannot exclude the possibility that inappropriate levels of stable FoxP3 expression during early thymic development could affect development of functional Treg and T effector cells. Further experiments will be necessary to determine if the function of Treg cells is compromised in APDS patients.

Many APDS patients, including Patient I in this study, have CMV viremia. It is known that IFN-γ production by CD4^+^ T cells is critical for controlling CMV infection (24). However, Bier et al. have recently shown that on average T_FH_ cells from APDS patients actually have a moderately increased percentage of CXCR3^+^ IFN-γ-producing cells compared to healthy controls (25). Unfortunately, Patient I was lost to follow up, but we examined cytokine production by CD4^+^ T cell blasts from Patients II.A and II.B. While IL-21 production was not significantly enhanced compared to control, there was a striking reduction of IFN-γ-producing cells Patient II.A and a moderate reduction in Patient II.B (Figure 3B). Furthermore, the percentage of cells expressing CXCR3, the chemokine receptor associated with Th1 cells, was not significantly enhanced in either CXCR5^+^PD-1^+^ T_FH_ cells or CXCR5^−^ non-T_FH_ cells (Figures 3C,D). We did note, however, an increase in the proportion of T_FH_ cells that expressed both CXCR3 and CCR6, the chemokine receptor associated with Th17 cells. These “double-positive” cells have been noted in other PIDs; they are generally able to help B cells, but not as well as CCR6-only T_FH_ cells (26). It is possible that the discrepancy between our results and those of Bier et al. is due to the nature of the APDS variants studied—a large majority of patients had the common E1021K variant in the catalytic domain of p110δ (25). Regardless, while enhanced T_FH_ differentiation appears to be universal among APDS patients, the cytokines produced by these T_FH_ cells appear to be variable, and the failure to control CMV in these patients points to a defect in Type I immunity.

**Figure 4:**
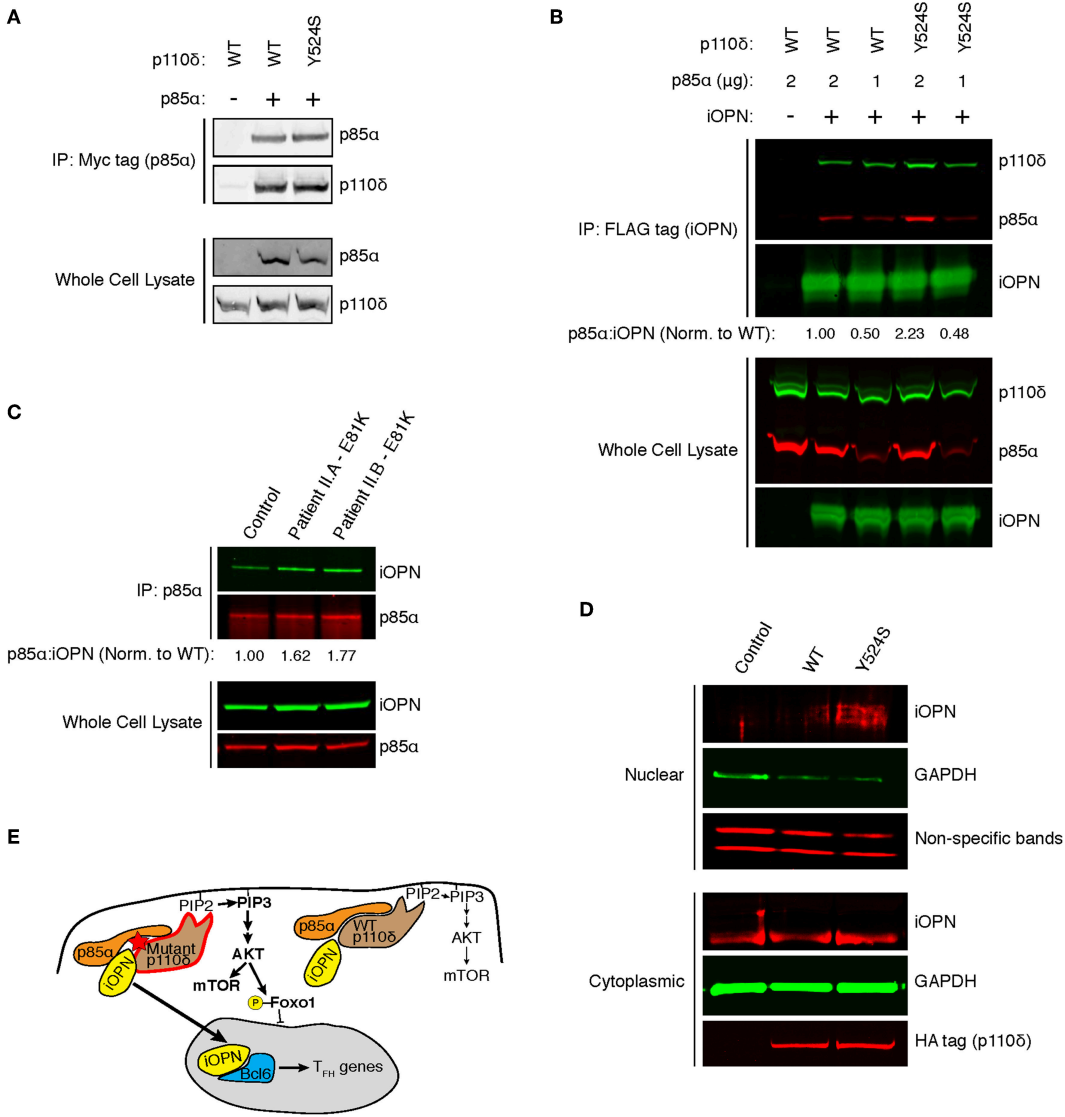
APDS variants enhance p85α-iOPN interactions. **(A)** Immunoprecipitation of WT or Y524S p110δ with p85α. 293T cells were co-transfected with HA-p110δ and Myc-p85α, and p85α was immunoprecipitated with an anti-Myc affinity gel. Results are representative of two experiments. **(B)** Co-immunoprecipitation of p85α with iOPN in the presence of WT or Y524S p110δ. 293T cells were co-transfected with WT or variant HA-p110δ, iOPN-FLAG and the indicated amount of Myc-p85α. iOPN was immunoprecipitated with an anti-FLAG affinity gel. Results are representative of two experiments. **(C)** Co-immunoprecipitation of iOPN with p85α from control and patient CD4^+^ T cell blasts. Results are representative of two experiments. **(D)** Intracellular OPN expression in nuclear and cytoplasmic fractions of transduced (GFP+) cells. CD4^+^ T cells from a healthy donor ere transduced with WT or Y524S p110δ. Blotting for GAPDH shows proper fractionation. Non-specific low molecular weight bands from the OPN blot show equal protein loading in the nuclear fractions. Results are representative of two experiments. **(E)** Model showing that enhanced interaction of iOPN with p85α in the presence of the variant p110δ drives T_FH_ differentiation.

In murine T cells, ICOS signaling results in a p85α-iOPN interaction, inducing T_FH_ differentiation, but whether these results hold for human T_FH_ has not been shown (14). We hypothesized that our patient's variant in the helical domain of p85α could disrupt inhibitory p110δ-p85α contacts, enhancing p85α-iOPN interactions. Unfortunately, Patient I was lost to follow-up, so we developed an *in vitro* system to investigate the effect of the Y524S variant on p85α-iOPN interactions. As previously shown for the pathogenic variants E525K and E1021K (2), immunoprecipitation experiments demonstrated that there was no difference in p110δ-p85α interactions for the Y524S variant (Figure 4A). To test for an enhancement of interactions between p85α and iOPN, we transfected 293T cells with plasmids encoding epitope-tagged iOPN, p85α, and either WT or variant p110δ and performed co-immunoprecipitation experiments. The interaction between iOPN and p85α was increased in the presence of the Y524S p110δ variant (Figures 4B, S1A). Notably, p110δ was also pulled down in a similar ratio to p85α, indicating the formation of a tri-molecular complex. To determine if the enhanced p85α-iOPN interaction was unique to the Y524S variant, we conducted immunoprecipitation experiments with CD4^+^ T cell blasts from the patients with an E81K variant. We saw enhanced co-immunoprecipitation of iOPN when p85α was pulled down (Figures 4C, S1B). This result confirms that pathogenic variants of p110δ enhance the interaction of iOPN with p85α.

Binding of iOPN to p85α results in translocation of iOPN into the nucleus where it stabilizes Bcl-6 (14). To determine if mutant p110δ induces nuclear translocation of iOPN, we transduced CD4^+^ T cells from healthy donors with WT or Y524S p110δ and examined nuclear and cytoplasmic fractions for the presence of iOPN. We found increased translocation of iOPN in the presence of the Y524S mutant p110δ (Figures 4D, S1C). The iOPN detected in the nucleus was of a slightly larger molecular weight than the bulk of iOPN found in the cytoplasm, indicative of phosphorylation. Notably, p85α preferentially interacts with phosphorylated iOPN in the cytoplasm and chaperones the phospho-protein into the nucleus (14).

## Discussion

Here, we have described a patient with a novel APDS variant, Y524S. The patient had excessive proportions of T_FH_ cells, both in the periphery and in lymph nodes. Notably, the patient had low numbers of circulating B cells, but her profound lymphadenopathy was successfully treated by B cell depletion. Our results suggest that the lymphadenopathy in APDS is the result of accumulation of germinal center B cells secondary to inappropriate T_FH_ differentiation. This exaggerated tendency may drive B cell lymphomas as well.

Co-immunoprecipitation experiments have shown that there is no difference in p110δ-p85α interactions when p110δ alleles containing APDS-causing variants are compared to WT [(2, 21) and Figure 4A]. However, hydrogen-deuterium exchange experiments have demonstrated that APDS variants in p110δ cause conformational changes that mimic or enhance the changes seen upon activation (27). Of note, increased hydrogen-deuterium exchange was seen at the helical-nSH2 interface when the E525K helical domain variant was present (27). The SH2 domain of p85α is thought to interact with a region of iOPN containing a phosphotyrosine (14). We hypothesize that the nSH2 domain is more accessible to iOPN when APDS variants are present, enhancing p85α-iOPN interaction and tipping the scales toward T_FH_ differentiation (Figure 4E). However, the presence of T_FH_ cells in patients with catalytic domain variants (11), indicate that multiple mechanisms are likely to enhance p85α-iOPN interactions in the presence of destabilizing APDS variants. Further experiments will be required to determine how the enhanced p85α-iOPN interactions and increased catalytic activity (and FOXO1 phosphorylation) seen with APDS variants synergize to promote T_FH_ differentiation.

The Y524S variant described here alters a potential phosphorylation site. Interestingly, the constitutively-active D816V variant of the receptor tyrosine kinase c-kit, which is found in leukemias and systemic mastocytosis, was shown to phosphorylate Y524 of p110δ in a murine B cell line (28). Although wild type c-kit could not phosphorylate p110δ, further research is needed to determine whether phosphorylation of Y524 is physiologically relevant. The enhanced iOPN-p85α interaction that we observed in the presence of the Y524S p110δ variant is most likely the result of increased accessibility of the nSH2 region of p85α by iOPN, and thus the role of Y524 as a phosphorylation site is unlikely to be related to T_FH_ differentiation. We cannot rule out other deleterious effects related to the loss of the phosphorylation site at Y524, although any effects would be unrelated to the lymphoproliferative aspects of the syndrome since phosphorylation at Y524 enhanced proliferation and survival in transformed cells.

Most reports describing APDS patients have focused the enhanced and unregulated lipid-kinase activity. Indeed, the primary treatment for APDS has been sirolimus (rapamycin) which inhibits mTOR downstream of hyperactive PI3K (4). Additionally, recent progress has been made treating APDS with the p110δ-specific kinase inhibitor leniolisib (29). Our results suggest that patients should also be monitored for alterations in T cell differentiation. These defects are likely to occur independently of the kinase activity of PI3K and may not improve with rapamycin or p110δ-specific inhibitor treatment.

## Materials and Methods

### Whole-Exome and Sanger Sequencing

Whole exome and Sanger sequencing were performed in the Baylor College of Medicine medical genetics lab.

### Molecular Modeling

The crystal structures of p110δ-p85α (5DXU), p110α-p85α (3HMM), and p110δ-p85α (5T27) were loaded in Coot to generate a model of p110δ-p85α that includes the loop bearing Y524 and the mutated Ser. Structural representation of the variant was made in PyMol.

### T Follicular Helper Differentiation

CD4^+^ T cells were isolated from PBMCs by immunomagnetic negative selection (EasySep; STEMCELL), or were purified from whole blood with RosetteSep (STEMCELL). For T_FH_ differentiation experiments, T cells were cultured in 96-well plates in complete T cell media (RPMI-1640 supplemented with 10% FBS, 10 mM HEPES, 2 mM L-Glutamine, and Pen/Step). The wells were pre-coated with the indicated concentrations of anti-CD3ε (clone OKT3; Biolegend) and 2 μg/mL ICOS-ligand-Fc (R&D Systems; 165-B7-100). Soluble anti-CD28 (clone CD28.2; Biolegend) was added at 1 μg/mL. At day 4, the T cells were harvested and stained as below.

### Generation of T Cell Blasts

*For the Treg experiments*: Peripheral blood mononuclear cells (PBMC) were isolated by density centrifugation using Ficoll-paque plus (GE Healthcare). The cells were plated in X-VIVO 15 medium supplemented with gentamycin (Lonza) and 5% pooled AB human serum (HS; Sigma-Aldrich). Phytohaemagglutinin (2 μg/mL; Sigma-Aldrich) and 30 u/mL IL-2 (Peprotech) were added at the beginning of the culture. The cell cultures were supplemented with 50 U/mL IL-2, 10 ng/mL IL-7 (R&D Systems), and 10 ng/mL IL-4 (R&D Systems). Cells were collected, washed with PBS, and restimulated with Dynabeads coated with anti-CD3 and anti-CD28 antibodies (Life Technologies) at a 1:20 bead:cell ratio in the presence of IL-2 (50 u/mL). After 72 h, the cells were collected, washed and stained. *For the ICCS and co-immunoprecipitation experiments*: CD4^+^ cells were purified from PBMC as above. Cells were stimulated with plate-bound anti-CD3ε (1 μg/mL) and soluble anti-CD28 (2 μg/mL). At day 3, cells were removed from anti-CD3ε coated wells and cultured in the presence of 100 U/mL IL-2. CD4^+^ blasts were used between day 7 and 12.

### Generation of Lentiviral Supernatants

Lenti-X 293T cells (Takara) were co-transfected (Lipofectamine 3000 reagent; ThermoFisher) with a lentiviral transfer plasmid containing WT or mutant PIK3CD (see below), the pCMV delta R8.2 packaging plasmid (Addgene #12263) and the pMD2.G envelope plasmid (Addgene #12259) at a 1:1:1 molar ratio. Supernatants were collected at 24 and 48 h post-transfection, pooled and pelleted by centrifugation using Lenti-X Concentrator (Takara). The resulting pellets were gently resuspended in T cell media and used immediately.

### T Cell Transduction

CD4^+^ T cells were isolated from the PBMC of deidentified, healthy donors as above. Cells were stimulated with Dynabeads at a 1:1 bead:cell ratio for 24 h prior to transduction. T cells were resuspended in lentiviral supernatants supplemented with 8 μg/mL polybrene. The cells were then plated on non-TC treated wells pre-coated with RetroNectin and centrifuged for 1 h at 1,200 × *g*. Lentiviral supernatants were replaced with fresh T cell media supplemented with 50 U/mL IL-2. GFP^+^ cells were sorted at day 5–7 post-transduction. Sorted cells were restimulated and expanded with Dynabeads prior to use. Cells maintained uniform levels of GFP expression while in culture.

### FACS Analysis

Freshly isolated PBMCs or cultured T cells were resuspended in FACS buffer (PBS with 2% FBS and 1 mM EDTA) and treated with Human TruStain FcX to block Fc receptors (Biolegend). Cells were stained with the following antibodies, all from Biolegend: anti-CD4 PerCP-Cy5.5 (clone RPA-T4), anti-ICOS Alexa Fluor 488 (clone C398.4A), anti-CXCR5 PE (clone J252D4), anti-CXCR3 PE-Cy7 (clone G025H7), anti CCR6 Alexa Fluor 488 (clone G034E3), and anti-PD-1 Alexa Fluor 647 (clone EH12.2H7). *For staining of Tregs*: cells were surface stained with anti-CD3 BV 605 (clone OKT3), anti-CD4 APC-Cy7 (clone RPA-T4), and anti-CD25 BV 421 (clone M-A251) (all from Biolegend), fixed and permeabilized with the FOXP3 Staining Buffer Set (ThermoFisher Scientific) and then stained with anti-FOXP3 Alexa Fluor 647 (clone 259D/C7; BD Biosciences).

### Intracellular Cytokine Staining

CD4^+^ T cell blasts were cultured in fresh media with or without 50 ng/mL PMA and 1 μM ionomycin (Sigma-Aldrich) for 4 h. Golgiplug (BD Biosciences) was added to all wells for the final 3 h of culture. Cells were stained with anti-CD4 BV 421(clone OKT4; Biolegend), fixed with 2% paraformaldehyde in PBS for 30 min at RT, and permeabilized with 0.5% saponin (Sigma-Aldrich) in FACS buffer for 10 min at RT. Cytokines were stained with anti-IFN-γ PE (clone 4S.B3; Biolegend) and anti-IL-21 Alexa Fluor 647 (clone 3A3-N2.1; BD Biosciences).

### Intracellular Phospho-Akt Staining

Frozen day 12 PHA blasts were thawed and cultured in complete T cell media supplemented with 50 U/mL IL-2 for 3 h. Cells were then either fixed directly with an equal volume of pre-warmed Cytofix buffer (BD Biosciences) or stimulated. For stimulation, cells were preincubated with 5 μg/mL anti-CD3ε and anti-CD28 on ice for 20 min and washed. Cells were stimulated with 20 μg/mL goat anti-mouse IgG (Jackson ImmunoResearch) for 10 min in complete T cell media and then fixed with pre-warmed Cytofix buffer. Cells were washed with FACS buffer and permeabilized with BD PhosFlow Perm Buffer III pre-chilled to −20°C and incubated for 30 min on ice. Cells were washed extensively and then stained with the following antibodies or appropriate isotype controls from Cell Signaling Technology: anti-pAkt (Ser473) Alexa Fluor 647 (clone D9E) and anti-pS6 (Ser240/244) Alexa Fluor 488 (clone D68F8).

### Immunoblot Analysis

Purified CD4^+^ T cells from freshly isolated PBMCs were pre-incubated with 5 μg/mL anti-CD3e and anti-CD28 on ice for 20 min, washed and resuspended in serum-free RPMI-1640. Cells were stimulated by adding goat anti-mouse IgG at a final concentration of 50 μg/mL and incubating at 37°C for the indicated times. The stimulation was stopped and cells were lysed by adding Bolt LDS sample buffer and sample reducing agent (ThermoFisher Scientific) directly to the tubes. Samples were separated on Bolt 4–12% Bis-Tris gels and transferred to PVDF membranes with the iBlot 2 system (ThermoFisher Scientific). Membranes were blocked with PBS containing 5% BSA and probed with the following antibodies: anti-pAkt-Ser473 (clone D9E; Cell Signaling Technology), anti-phosphotyrosine (4G10; EMD Millipore), and anti-b-actin-HRP (clone C4; Santa Cruz Biotechnology).

### Nuclear/Cytoplasmic Fractionation

Cells were washed in ice-cold PBS and fractions were prepared using a Nuclear and Cytoplasmic Extraction Kit, following the manufacturers instructions (G-Biosciences). Western blotting was conducted as above, using the following antibodies: anti-Osteopontin (ab8448; Abcam), anti-GAPDH (clone G-9; Santa Cruz Biotechnology), and anti-HA tag (clone C29F4; Cell Signaling Technology).

### Constructs and Co-immunoprecipitation

The entire coding sequences of human *PIK3R1* (encoding p85α) and *PIK3CD* (encoding p110δ) were cloned from cDNA (Open Biosystems BC094795 and BC132921, respectively) into pcDNA 3.1+ (Invitrogen) or murine stem cell virus (MSCV) vectors, respectively. A c-Myc tag was cloned onto the N-terminus of p85α, and an HA tag was cloned onto the N-terminus of p110δ and variants were introduced with PCR-based site-directed mutagenesis. For lentiviral constructs, HA-tagged WT or mutant *PIK3CD* was upstream of the EF1α promoter in the EF.PGK.GFP plasmid (Addgene #17618). The coding sequence of human *SPP1* (Osteopontin, Open Biosystems, BC017387) minus bases corresponding to the 16 amino acid N-terminal signal sequence was cloned into pcDNA3.1+, and a DYKDDDDK tag was attached to the C-terminus.

HEK-293T cells were co-transfected (Lipofectamine LTX reagent; ThermoFisher) with a total of 6 μg of plasmid DNA; 2 μg each of plasmids encoding p85α, p110δ, and iOPN. In some experiments, only p85α and p110δ were transfected. Forty-eight hours after transfections, cells were harvested, washed extensively with PBS and crosslinked with 2.5 mM dithiobis(succinimidyl propionate) (DSP) in PBS for 20 min at RT. The reaction was stopped with the addition of 1 M Tris-HCl to a final concentration of 10 mM and the cells were washed with ice-cold PBS and lysed in RIPA buffer containing HALT protease inhibitors (ThermoFisher). Insoluble material was removed by centrifugation and lysates were incubated with anti-DYKDDDDK (FLAG) affinity gel (clone L5; Biolegend) overnight at 4°C. Alternatively, an anti-Myc affinity gel (9E10; Biolegend) was used to pull down p85α. Prior to use, affinity gels were blocked for >2 h with 293T lysates from untransfected cells. After extensive washing with RIPA buffer, the affinity gel was incubated with Bolt LDS sample buffer and NuPAGE Sample Reducing Agent to release precipitated protein and reduce DSP crosslinks. Whole cell lysates and immunoprecipitates were separated on gels as above and transferred to nitrocellulose membranes. Membranes were probed with anti-Myc tag (clone 9B11), anti-HA tag (clone C29F4), and polyclonal anti-FLAG tag (Cat# 2368) all from Cell Signaling Technology. Fluorescent secondary antibodies against mouse and rabbit were from LI-COR and blots were imaged with an Odyssey Infrared Imaging System (LI-COR).

*For the co-immunoprecipitation experiments with T cell blasts*: Cells were cross-linked with DSP and lysed as above. Lysates were pre-cleared with Protein A/G-agarose (Santa Cruz Biotechnology) for 1 h at 4°C. p85α was immunoprecipitated with anti-p85-agarose (16–107; Millipore Sigma) overnight at 4°C. Immunoprecipitates were processed as above and membranes were probed with anti-p85α (ABS233; Millipore Sigma) and anti-Osteopontin (clone 53; Abcam).

### Study Approval

Research on human subjects was performed after written informed consent was obtained and under the auspices of the Stanford University and UCLA Institutional Review Boards.

## Ethics Statement

This study was carried out after written informed consent from all subjects. All subjects gave written informed consent in accordance with the Declaration of Helsinki. The protocol was approved by the Stanford University and UCLA Institutional Review Boards.

## Author Contributions

MB provided patient care and obtained IRB approval. RO prepared histology images. MB, TT, and RB designed the research. TT and LP conducted experiments and analyzed data. MB made the molecular model. MB and TT wrote the manuscript.

### Conflict of Interest Statement

The authors declare that the research was conducted in the absence of any commercial or financial relationships that could be construed as a potential conflict of interest.
