# Supplementary figures and images for: Case Study: Mechanism for Increased Follicular Helper T Cell Development in Activated PI3K Delta Syndrome
Source: Front Immunol. 2019 Apr 12;10:753. doi: 10.3389/fimmu.2019.00753 (PMC6473200; doi:10.3389/fimmu.2019.00753)

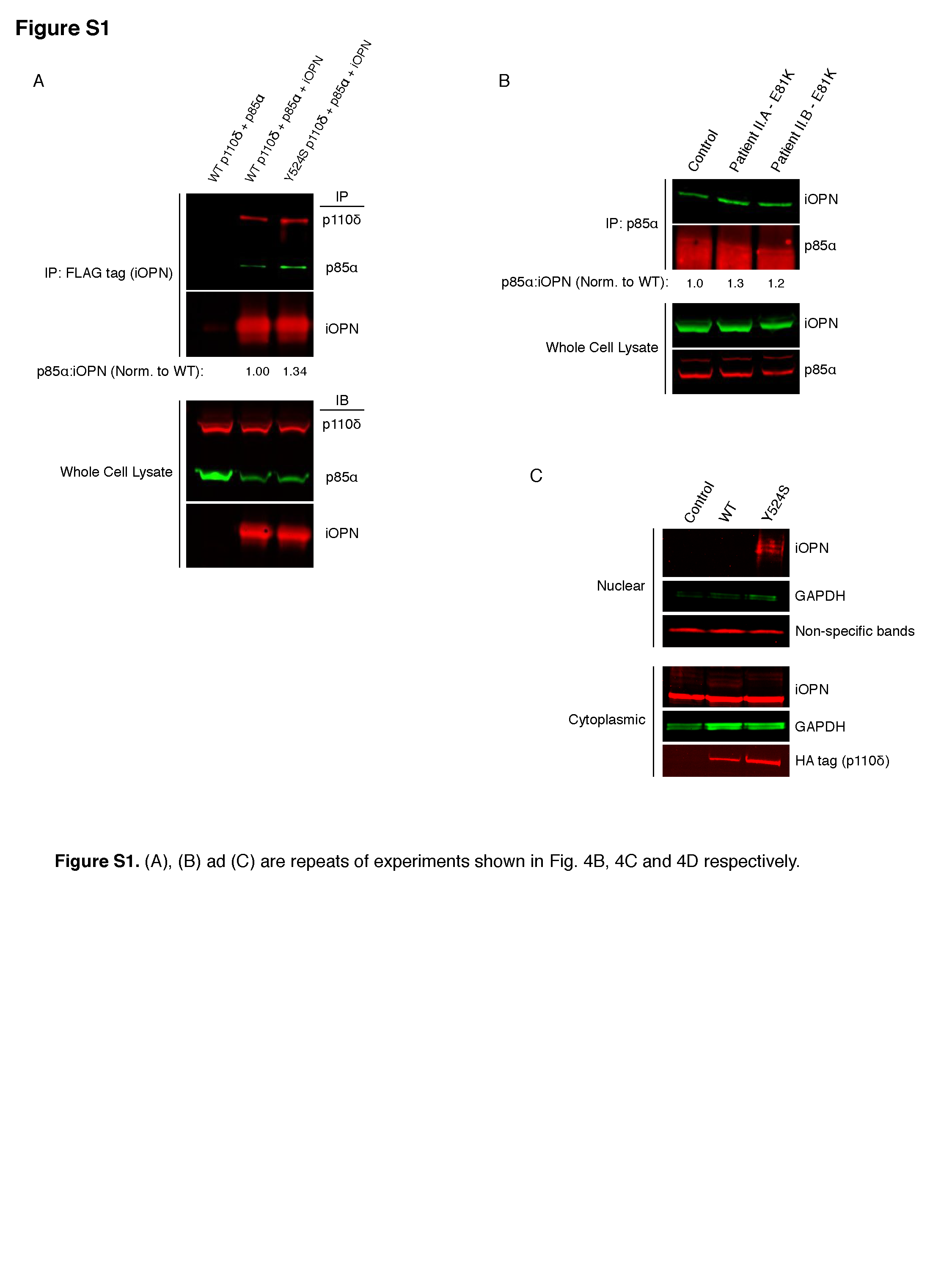

Supplement: Supplementary file 1 [file Image_1.TIFF]

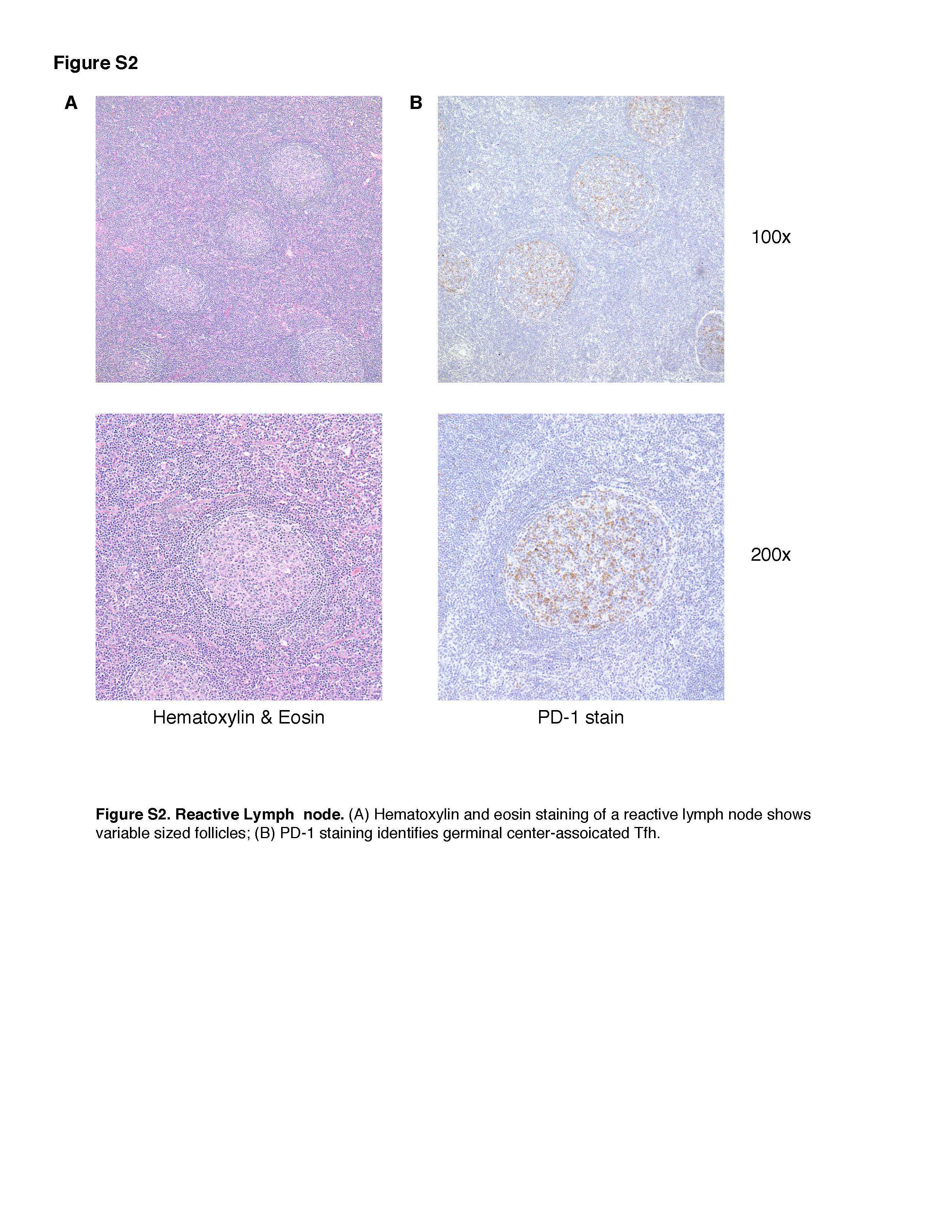

Supplement: Supplementary file 2 [file Image_2.TIFF]
